# Replantation of a Permanent Tooth With a Curved Root From Infected Tooth Germ: A Case Report With a Four-Year Follow-Up

**DOI:** 10.7759/cureus.72967

**Published:** 2024-11-04

**Authors:** Shunsuke Sawada, Yuki Sakamoto, Hiroki Shikita, Yuka Kojima

**Affiliations:** 1 Department of Dentistry and Oral Surgery, Kansai Medical University Hospital, Hirakata, JPN; 2 Department of Oral Surgery, Kansai Medical University Medical Center, Moriguchi, JPN

**Keywords:** impacted tooth, intentional replantation, oral and maxillofacial surgery, replantation, tooth germ

## Abstract

Intentional reimplantation has long been supported by many clinicians as a last resort before tooth extraction. With the accumulation of data and the development of techniques, the survival rate of reimplantation has increased. However, although there have been many reports and studies on replantation for common causes such as root fracture, root resorption, inadequate root canal treatment, perforation, and apical periodontitis, the method of replantation in unusual cases is not yet clear, and many cases result in tooth extraction. In this report, we describe a special case in which a patient requested the replantation of an infected immature tooth with an abnormally curving root. The patient, a seven-year-old boy, sustained a bruise on his anterior maxillary tooth due to a fall at the age of one year. He had been symptom-free since then, but he came to see his dentist with a chief complaint of pain in the left maxillary gingiva. An immature permanent tooth was retrogradely impacted under the left maxillary deciduous central incisor and bone permeation was observed around the tooth. After referral to our department, the deciduous incisor was extracted and drained of pus as the first step. In the second step, a permanent central incisor was replanted at the patient's request. After four years of observation of the replanted teeth, the patient's prognosis was satisfactory. The tooth was replanted under general anesthesia. This is the first report of a permanent tooth with a curved inverted, impacted, or incomplete root infection, and the patient's progress was good after over four years of follow-up.

## Introduction

Many successful cases of avulsed tooth replantation have been reported [1]. There have also been reports of teeth being intentionally extracted and replanted immediately [2,3]. However, prognostic factors are yet to be elucidated, and tooth replantation is not always successful. Tooth replantation under unpredictable and irregular conditions, such as an impacted tooth with infection and a right-angled curving root, has low predictability and results in early extraction. However, extracting permanent teeth in childhood should be avoided considering potential future problems, as it could place a heavy burden on the patient. We replanted an immature impacted permanent tooth with abnormal root growth caused by infection of the tooth germ initially believed to have a poor prognosis. We monitored the progress for four years and noted a good prognosis.

## Case presentation

**Figure 1:**
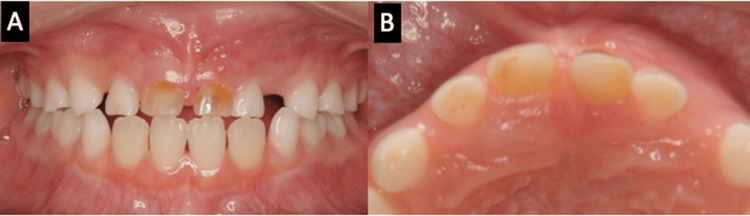
Intraoral view at the first visit (A-B). Discoloration on the crowns of both deciduous maxillary incisors is observed. Redness and swelling are observed in the distal gingiva of the left deciduous maxillary incisor.

We report a case of a seven-year-old boy who injured his deciduous maxillary anterior teeth from a fall at the age of one year. He underwent root canal treatment for deciduous maxillary central incisors at the dental office. Thereafter, he remained symptom-free, but approximately six years later, he complained of pain in his left deciduous maxillary central incisor and visited a dental clinic. The patient was referred to our department for further investigation. Initial examination revealed mild movement of the left deciduous maxillary central incisor. Redness and swelling were also observed in the gingiva of the left deciduous maxillary incisor (Figure 1).

**Figure 2:**
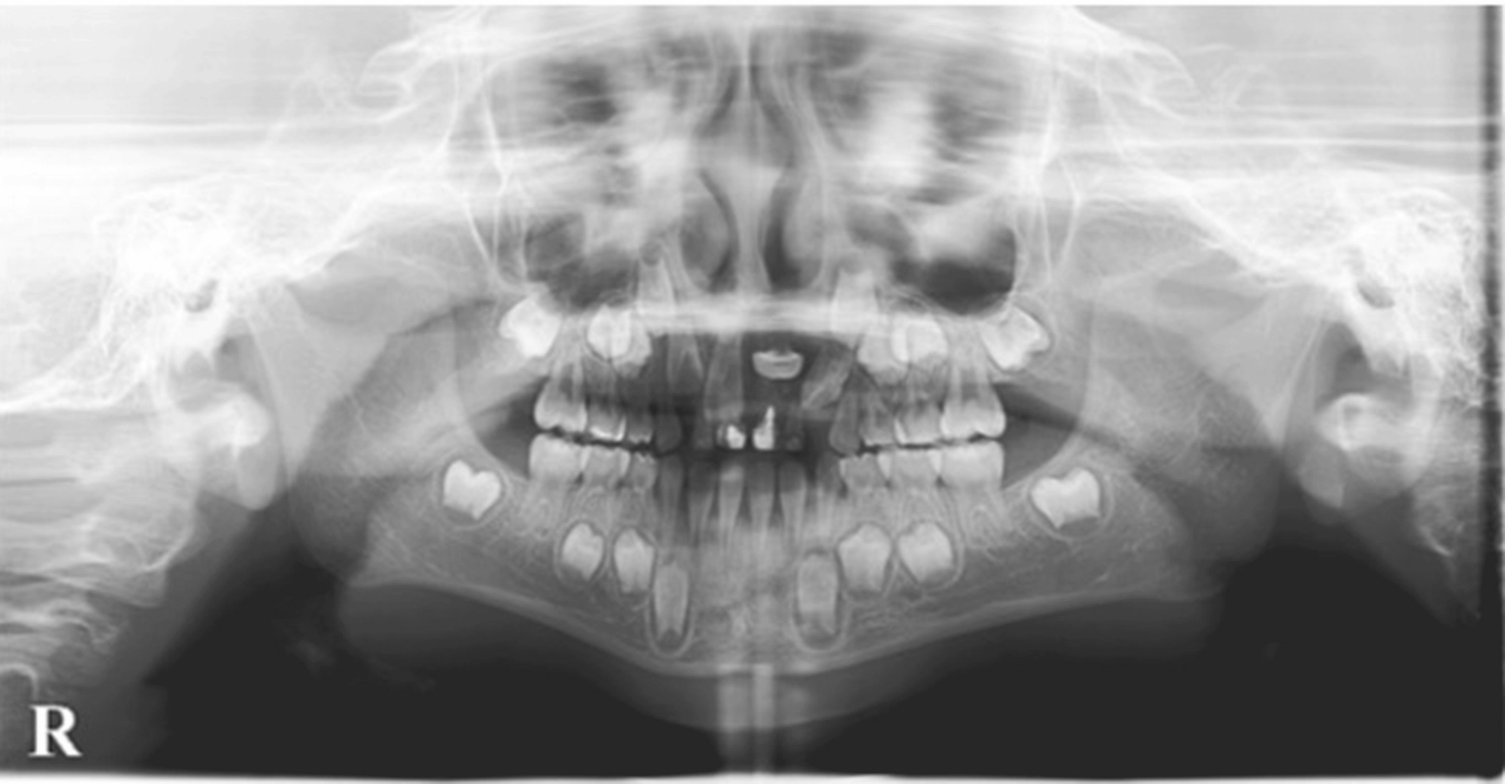
Panoramic radiograph taken at the first visit. The left maxillary central incisor is invertedly impacted at the root apex of the left deciduous maxillary incisor, and a circular radiographic image surrounding the crown and root of the left maxillary central incisor is observed.

Regarding radiographic findings, the left maxillary central incisor, a permanent tooth, was inverted and impacted at the root apex of the left deciduous maxillary central incisor. Bone resorption was observed around the crown of an impacted central incisor on a panoramic X-ray (Figure 2). The patient did not have a relevant medical history or a history of oral medications or allergies.

**Figure 3:**
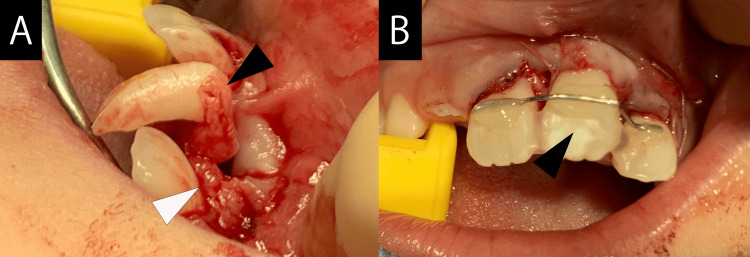
Intraoperative and post-replant images. (A) Roots with curved right angles are confirmed (black arrow). Granulation tissue is observed (white arrow). (B) The treatment was performed with a conscious effort to shorten the time required for extraoral operations. The replanted tooth was fixed with a wire (black arrow).

**Figure 4:**
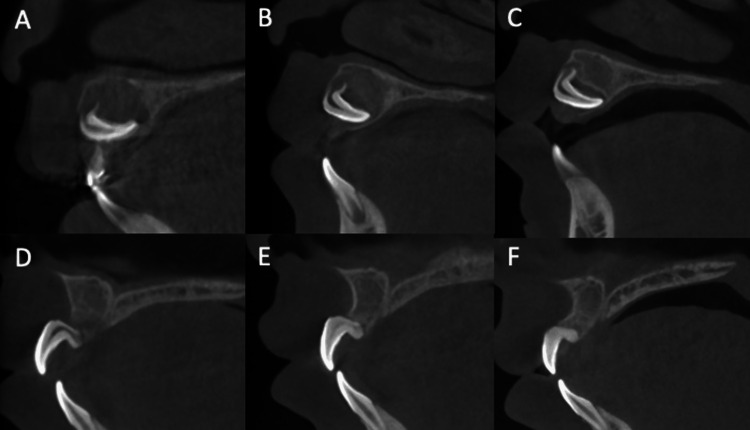
Cone-beam computed tomography scan. (A) At the first visit. (B) Three months after deciduous tooth extraction. (C) Seven months after deciduous tooth extraction. (D) Three months after replantation. (E) Seven months after replantation. (F) Four years after replantation. Before replantation in A–C, root growth is observed, but there are no changes in the impacted position and direction of the left maxillary central incisor. After replantation in D–E, the formation of surrounding alveolar bone and narrowing of the pulp cavity are observed.

**Figure 5:**
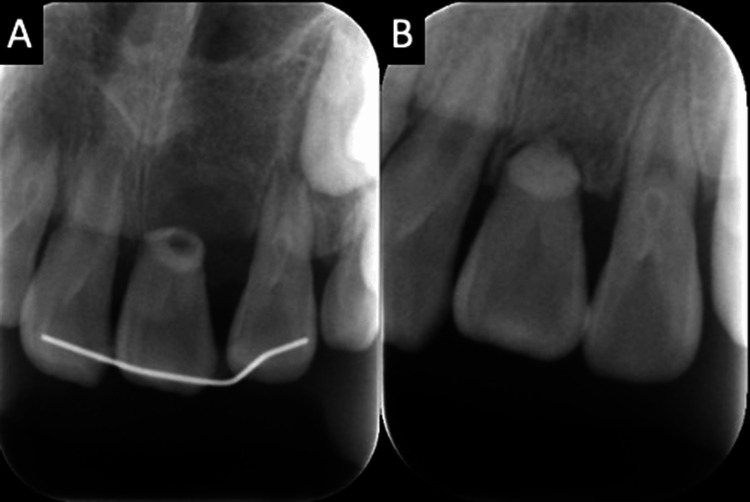
Dental radiograph. (A) Immediately after surgery. (B) Two and a half years later.

**Figure 6:**
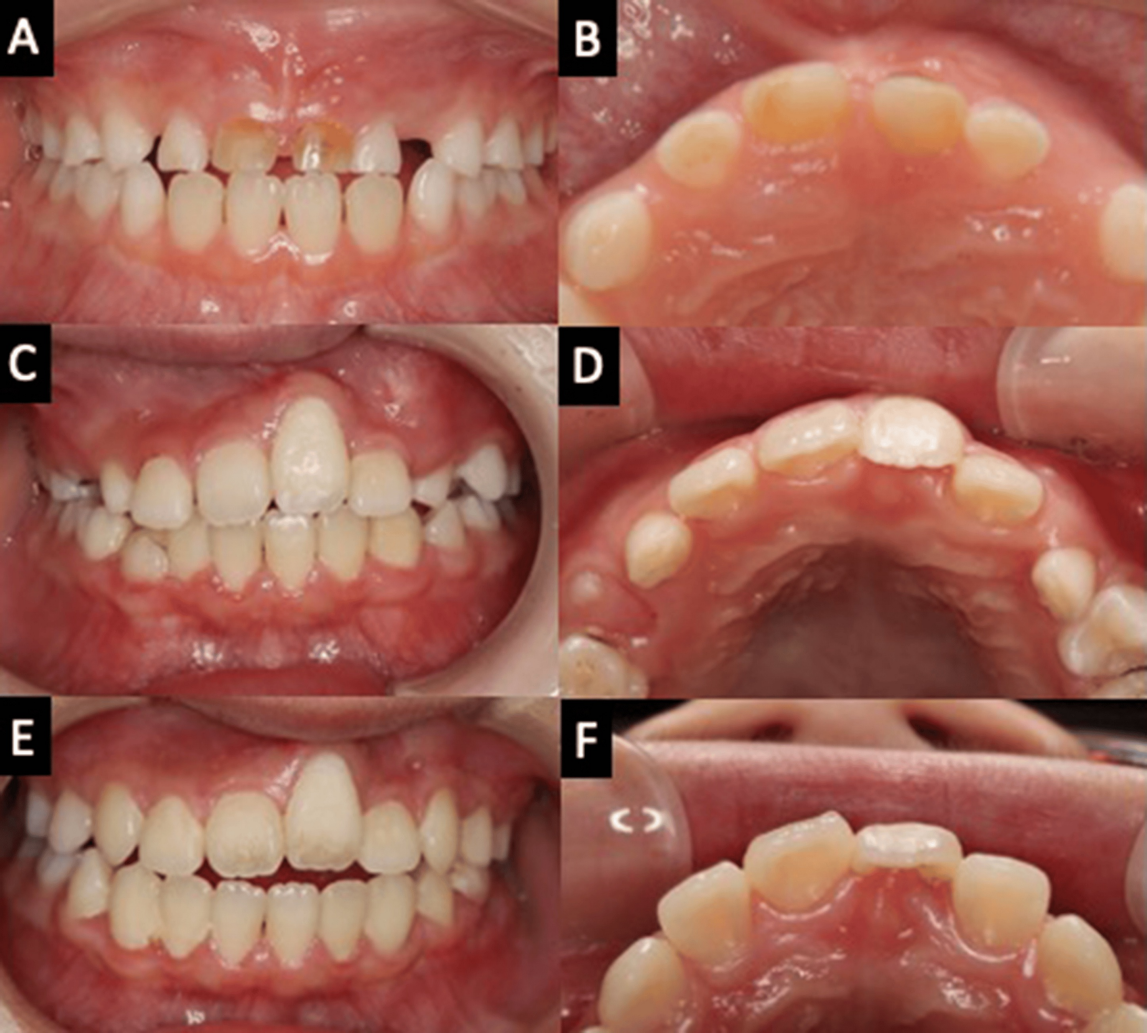
Intraoral photos over time. (A, B) At the first visit. (C, D) Two years after surgery. (E, F) Four years after surgery.

The treatment consisted of extracting the bilateral deciduous maxillary central incisors and left deciduous maxillary lateral incisor. Drainage of pus was also encouraged during the same procedure. Three months after extraction of the deciduous tooth and pus drainage at the same time, the bone resorption area, including the crown, showed a reduction, but no change in the position or orientation of the impacted permanent tooth was observed. The patient expressed a desire to avoid permanent tooth extraction. After consulting the patient’s family and obtaining their consent, we decided to perform replantation under general anesthesia. We explained the possibility of future extraction and complications, such as root resorption, and the left maxillary central incisor was replanted and fixed with a wire (Figure 3). The wire fixation was removed three months later. Four years later, gingival recession was observed; however, no signs of movement or infection were observed, and the patient progressed well (Figures 4-6).

Ethics approval was exempted, but the study was conducted in accordance with the Declaration of Helsinki. Informed consent was obtained from the patient.

## Discussion

In a systematic review, Wang et al. reported that long-term success and healing rate of intentional replantation depends on a short extraoral manipulation time, reduced pocket depth, tooth type, type of material of the root canal, and reduced damage to the root [4]. Other reports have indicated that immature teeth are appropriate candidates for autografting, with excellent survival and success rates [5]. Shortening extraoral time plays a role in the success of replantation [6-8]. We minimized the time the replanted tooth was out of the mouth during surgery and performed manipulation gently while considering root damage carefully; this contributed to successful replantation.

Root canal filling is usually performed during intentional replantation [9]. However, pulp cavity narrowing is common after replantation [10]. We planned to perform root canal treatment if symptoms appear after replantation or tooth extraction if the prognosis is poor. However, as the pulp cavity narrowed over time, the patient had no root periapical lesions or other subjective symptoms, and we did not perform root canal treatment.

Ankylosis may occur after successful replantation, particularly if the teeth retain sufficient postoperative masticatory function and serve as permanent teeth. In this case, ankylosis could not be ruled out. Cone-beam computed tomography is useful in confirming ankylosis, although it remains challenging to establish a definitive diagnosis even with imaging [11-13]. Although the possibility of ankylosis could not be ruled out, we did not observe any symptoms. Even if the replanted tooth is ankylosed, it is considered to perform its full function as a permanent tooth, providing space and maintaining masticatory function.

Chamberlin and Goerig [14] used the following criteria to evaluate replanting: teeth remain fixed in the sockets without residual inflammation; adequate masticatory function without discomfort; teeth showed no pathological mobility; absence of pathological conditions on radiography; normal lamina dura appearance on radiography; radiographs showing further tooth growth; and normal sulcus depth, gingival contour, and gingival color. In the present case, four years have passed since replantation, and almost all criteria are satisfied.

Patients should be fully informed about the possibility of common post-reimplantation contingencies (e.g., root resorption and pulpal necrosis), and a trusting relationship should be established. This relationship extends beyond the initial recognition of a contingency and involves following all postoperative instructions and attending regular hospital visits.

The patient had a curved permanent tooth with a history of infection. Generally, impacted permanent teeth with a history of infection and curved roots are considered for extraction. However, in this case, tooth replantation was performed in a difficult condition at the patient’s request. The replanted tooth was preserved under a stable condition for four years. Therefore, we believe that replantation should be considered under various circumstances in a trusting relationship with the patient.

Pisano et al. described data from 106 cases in a systematic review of replanted teeth [15]. Of these 106 cases, only one case was successfully replanted with the tooth completely included in the bone resorption radiograph. Furthermore, the root morphology was normal and the follow-up period was 12 months [16], so this was not a long-term case. Therefore, this report is a very rare case of success.

Although the treatment was successful, there are limitations to this report. As teeth in poor condition, such as in this case, are not usually replanted, the number of cases will be limited. Since conducting randomized controlled trials for such cases is difficult, reporting many cases in the form of case reports is important.

## Conclusions

In conclusion, a tooth with an inverted, impacted, and incomplete root that was curved because of infection was successfully replanted, and the patient's progress was satisfactory during four years of follow-up.

To the best of our knowledge, this is the first case report with a four-year follow-up of the replantation of a permanent tooth from an infected germ with a curved root. In the past reports, there have been no cases similar to our report, which makes this report very valuable. As shown in this report, replantation should be considered as one of the main treatment options under severe conditions of the tooth.
